# A new species of the Music frog *Nidirana* (Anura, Ranidae) from Guizhou Province, China

**DOI:** 10.3897/zookeys.904.39161

**Published:** 2020-01-16

**Authors:** Gang Wei, Shi-Ze Li, Jing Liu, Yan-Lin Cheng, Ning Xu, Bin Wang

**Affiliations:** 1 Biodiversity Conservation Key Laboratory, Guiyang College, Guiyang, 550002, China Guiyang College Guiyang China; 2 CAS Key Laboratory of Mountain Ecological Restoration and Bioresource Utilization and Ecological Restoration Biodiversity Conservation Key Laboratory of Sichuan Province, Chengdu Institute of Biology, Chinese Academy of Sciences, Chengdu 610041, China Chengdu Institute of Biology, Chinese Academy of Sciences Chengdu China; 3 Department of Food Science and Engineering, Moutai Institute, Renhuai 564500, China Moutai Institute Renhuai China

**Keywords:** Call, molecular phylogenetic analyses, morphology, *Nidirana yeae* sp. nov., taxonomy

## Abstract

The Music frog genus *Nidirana* is widely distributed in East and South Asia. Here, a new species of the genus is described from southwestern China. Phylogenetic analyses based on the mitochondrial 16S rRNA and COI gene sequences supported the new species as a clade closely related to *N. leishanensis*, *N. hainanensis*, *N. chapaensis*, *N. daunchina*, and *N. yaoica*. The new species could be distinguished from its congeners by a combination of the following characters: body of medium size (SVL 41.2–43.5 mm in males and 44.7 mm in female); lateroventral groove only present on toes; relative finger lengths: II < IV < I < III; three metatarsal tubercles on palm; heels overlapping when hindlimbs flexed at right angles to axis of body; tibiotarsal articulation reaching the level of eye when leg stretched forward; a pair of subgular internal vocal sacs at corners of throat in male; nuptial pad present on the inner side of base of fingers I in breeding male; tadpole labial tooth row formula with 1:1+1/1+1:2; in males, the advertisement call contains two kinds of notes and one call contains 2–6 repeated regular notes.

## Introduction

The Music frogs of the genus *Nidirana* Dubois, 1992 are widely distributed in East and Southeast Asia, from Japan westwards to southern China, and southwards to northern Thailand, northern Vietnam, and Laos (Frost 2019). Systematic arrangements of the group have been controversial for a long time (Dubois 1992; Chen et al. 2005; Frost et al. 2006; Fei et al. 2009, 2010, 2012; Chuaynkern et al. 2010). Lyu et al. (2017) confirmed it as a distinct genus based on comprehensive species sampling with molecular, morphological, and bioacoustics evidence. To date, *Nidirana* contained ten species: the type species *N. okinavana* (Boettger, 1895) occurring from Yaeyama of southern Ryukyu, and eastern Taiwan Island; *N. adenopleura* (Boulenger, 1909) from Taiwan Island to south-eastern mainland China; *N. hainanensis* (Fei, Ye & Jiang, 2007) from Diaoluo Mountain of Hainan Island of China; *N. daunchina* (Chang, 1933) from southwestern China; *N. pleuraden* (Boulenger, 1904) from southwestern China; *N. chapaensis* (Bourret, 1937) from the north-eastern Indochinese peninsula to south-eastern Yunnan Province, China; *N. lini* (Chou, 1999) from southern Yunnan Province, China, north-western Vietnam and Thailand; *N. nankunensis* (Lyu, Zeng, Wang, Lin, Liu & Wang, 2017) from Nankun Mountain, Guangdong Province, China; *N. leishanebsis* (Li, Wei, Xu, Cui, Fei, Jiang, Liu & Wang, 2019) from Leishan Mountain, Guizhou Province, China; and *N. yaoica* (Lyu, Mo, Wan, Li, Pang & Wang, 2019) from Dayao Mountain, Guangxi Province, China.

In all *Nidirana* species, *N. adenopleura* and *N. daunchina* were reported to have the widest distributional ranges in southwestern China and south-eastern China, respectively (Fei et al. 2009, 2012). Recently, two species (*N. yaoica* and *N. leishanebsis*) were recognised from two populations which had been identified as *N. adenopleura* although they were not phylogenetically sister taxa to *N. adenopleura* (Lyu et al. 2019a; Li et al. 2019a). As well, it is expected that there are cryptic species in populations being recognised as *N. daunchina* in its wide distributional range. Wu et al. (1986) found that the population classified as *N. adenopleura* from Suiyang County, Guizhou Province, China (later classified as *N. daunchina* by Fei et al. 2009) had some morphological differences with the population of the species from its type locality (E’mei Mountain, Sichuan Province, China). Hence, deeper investigations using molecular phylogenetic approaches are necessary to evaluate the taxonomic status of these populations.

In recent years, we carried out a series of biodiversity surveys in Tongzi County, Guizhou Province, China, and collected eleven specimens of *Nidirana*. Molecular phylogenetic analyses, morphological comparisons, and bioacoustics comparisons indicated the specimens as an unnamed species of *Nidirana*. We describe it herein as a new species.

## Materials and methods

### Specimens

**Figure 1.**
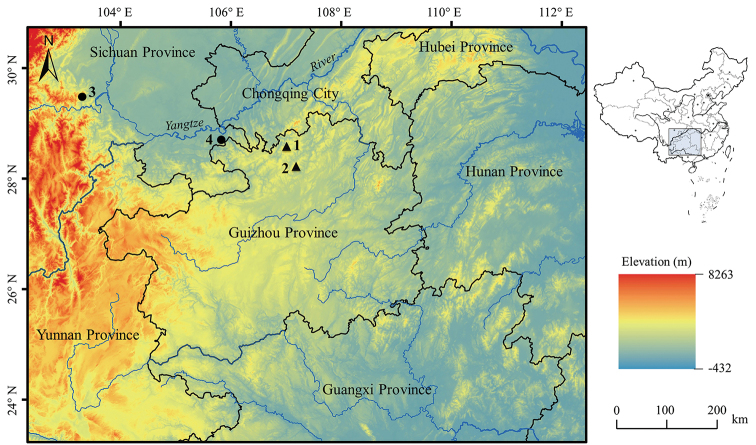
Type locality of *Nidirana yeae* sp. nov. and sampling localities of *N. daunchina*. **1**, the type locality of *Nidirana yeae* sp. nov., Huanglian town, Tongzi County, Guizhou Province, China; **2**, Kuankuoshui National Nature Reserve, Suiyang County, Guizhou Province, China as the potential distribution area deduced from Wu et al. (1986); **3**, the type locality of *N. daunchina*, E’mei Mountain, Sichuan Province, China; **4**, Hejiang County, Sichuan Province, China.

Nine adult males, one adult female, and one tadpole of the new species were collected from Huanglian Town, Tongzi County, Guizhou Province, China from 2015 to 2019 (for voucher information see Table 1, Fig. 1, Suppl. material 1: Table S1). After taking photographs, the animals were euthanised using isoflurane, and the specimens were then fixed in 10 % buffered formalin. Tissue samples were taken and preserved separately in 95 % ethanol prior to fixation. Specimens were deposited in Chengdu Institute of Biology, Chinese Academy of Sciences (**CIB, CAS**).

**Table 1.** Information for samples used in molecular phylogenetic analyses in this study.

ID	Species	Locality (* the type locality)	Voucher number	16S	CO1
1	*Nidirana yeae* sp. nov.	*Huanglian Town, Tongzi County, Guizhou Province, China	CIBTZ20190608004	MN295227	MN295233
2	*Nidirana yeae* sp. nov.	*Huanglian Town, Tongzi County, Guizhou Province, China	CIBTZ20190608005	MN295228	MN295234
3	*Nidirana yeae* sp. nov.	*Huanglian Town, Tongzi County, Guizhou Province, China	CIBTZ20190608019	MN295229	MN295235
4	*Nidirana yeae* sp. nov.	*Huanglian Town, Tongzi County, Guizhou Province, China	CIBTZ20190608006	MN295230	MN295236
5	*Nidirana yeae* sp. nov.	*Huanglian Town, Tongzi County, Guizhou Province, China	CIBTZ20160714016	MN295231	MN295237
6	*Nidirana yeae* sp. nov.	*Huanglian Town, Tongzi County, Guizhou Province, China	CIBTZ20190608003	MN295232	MN295238
7	*Nidirana daunchina*	*Emei Mountain, Sichuan Province, China	SYS a004595	MF807823	MF807862
8	*Nidirana daunchina*	*Emei Mountain, Sichuan Province, China	CIB2011081603	MK293821	MK293839
9	*Nidirana daunchina*	*Emei Mountain, Sichuan Province, China	CIB2011081601	MK293819	MK293837
10	*Nidirana daunchina*	*Emei Mountain, Sichuan Province, China	SYS a004594	MF807822	MF807861
11	*Nidirana daunchina*	*Emei Mountain, Sichuan Province, China	CIB2011081602	MK293820	MK293838
12	*Nidirana daunchina*	*Emei Mountain, Sichuan Province, China	CIB20110629001	MK293822	MK293840
13	*Nidirana daunchina*	Hejiang County, Sichuan Province, China	SYS a004930	MF807824	MF807863
14	*Nidirana daunchina*	Hejiang County, Sichuan Province, China	SYS a004931	MF807825	MF807864
15	*Nidirana daunchina*	Hejiang County, Sichuan Province, China	SYS a004932	MF807826	MF807865
16	*Nidirana yaoica*	*Daoyao Mountain, Guangxi Zhuang Autonomous Region, China	SYS a007009	MK882271	MK895036
17	*Nidirana yaoica*	*Daoyao Mountain, Guangxi Zhuang Autonomous Region, China	SYS a007011	MK882272	MK895037
18	*Nidirana yaoica*	*Daoyao Mountain, Guangxi Zhuang Autonomous Region, China	SYS a007012	MK882273	MK895038
19	*Nidirana yaoica*	*Daoyao Mountain, Guangxi Zhuang Autonomous Region, China	SYS a007013	MK882274	MK895039
20	*Nidirana yaoica*	*Daoyao Mountain, Guangxi Zhuang Autonomous Region, China	SYS a007014/CIB 110013	MK882275	MK895040
21	*Nidirana yaoica*	*Daoyao Mountain, Guangxi Zhuang Autonomous Region, China	SYS a007020	MK882276	MK895041
22	*Nidirana yaoica*	*Daoyao Mountain, Guangxi Zhuang Autonomous Region, China	SYS a007021	MK882277	MK895042
23	*Nidirana yaoica*	*Daoyao Mountain, Guangxi Zhuang Autonomous Region, China	SYS a007022	MK882278	MK895043
24	*Nidirana chapaensis*	*Sapa, Lao Cai, Vietnam	ROM 28070	AF206460	/
25	*Nidirana chapaensis*	*Sapa, Lao Cai, Vietnam	1999.5871	KR827710	/
26	*Nidirana chapaensis*	Gia Lai, Vietnam	AMSR176027	KU840598	/
27	*Nidirana chapaensis*	*Sapa, Lao Cai, Vietnam	T2483/2000.4850	KR827711	KR827711
28	*Nidirana hainanensis*	*Diaoluo Mountain, Lingshui County, Hainan Province, China	CIB20110629003	MK293807	MK293825
29	*Nidirana leishanensis*	*Leigong Mountain, Leishan County, Guizhou Province, China	CIBLS20150627003	MK293810	MK293828
30	*Nidirana lini*	*Jiangcheng County, Yunnan Province, China	SYS a003967	MF807818	MF807857
31	*Nidirana adenopleura*	*New Taipei City, Taiwan Province, China	UMMZ 189963	DQ283117	/
32	*Nidirana adenopleura*	Nanping City, Fujian Province, China	SYS a005911	MF807844	MF807883
33	*Nidirana okinavana*	*Iriomote Island, Okinawa, Japan	/	NC022872	NC022872
34	*Nidirana nankunensis*	*Nankun Mountain, Guangdong Province, China	SYS a003618	MF807828	MF807867
35	*Nidirana pleuraden*	Gaoligong Mountain, Yunnan Province, China	SYS a003775	MF807816	MF807855
36	*Babina holsti*	*Okinawa, Japan	/	NC022870	NC022870
37	*Babina subaspera*	*Amami Island, Kagoshima, Japan	/	NC022871	NC022871
38	*Odorrana margaretae*	China	HNNU1207003	NC024603	/

### Molecular data and phylogenetic analyses

Four male specimens, one female specimen, and one tadpole of the new species were included in the molecular analyses (for voucher information see Table 1). For phylogenetic comparisons, the corresponding sequences for other *Nidirana* species, two *Babina* species, and one *Odorrana margaretae* for which comparable sequences were available were downloaded from GenBank (Table 1). *Odorrana margaretae* was used as the outgroup following previous studies (Lyu et al. 2017, 2019a; Li et al. 2019a).

Total DNA was extracted using a standard phenol-chloroform extraction protocol (Sambrook et al. 1989). Two fragments of the mitochondrial 16S rRNA and cytochrome oxidase subunit I (COI) genes were amplified. For 16S, the primers P7 (5’-CGCCTGTTTACCAAAAACAT-3’) and P8 (5’-CCGGTCTGAACTCAGATCACGT-3’) were used following Simon et al. (1994), and for COI, Chmf4 (5’-TYTCWACWAAYCAYAAAGAYATCGG-3’) and Chmr4 (5’-ACYTCRGGRTGRCCRAARAATCA-3’) were used following Che et al. (2012). Gene fragments were amplified under the following conditions: an initial denaturing step at 95 °C for 4 min; 36 cycles of denaturing at 95 °C for 30 s, annealing at 52 °C (for 16S)/47 °C (for COI) for 40 s and extending at 72 °C for 70 s. Sequencing was conducted using an ABI3730 automated DNA sequencer in Shanghai DNA BioTechnologies Co., Ltd. (Shanghai, China). New sequences were deposited in GenBank (for GenBank accession numbers see Table 1).

Sequences were assembled and aligned using the Clustalw module in BioEdit v. 7.0.9.0 (Hall 1999) with default settings. Alignments were checked by eye and revised manually if necessary. For phylogenetic analyses of mitochondrial DNA, the dataset concatenated with 16S and COI gene sequences. To avoid under- or over-parameterisation (Lemmon and Moriarty 2004; McGuire et al. 2007), the best partition scheme and the best evolutionary model for each partition were chosen for the phylogenetic analyses using PARTITIONFINDER v. 1.1.1 (Robert et al. 2012). In this analysis, 16S gene and each codon position of COI gene were defined, and Bayesian Inference Criteria was used. As a result, the analysis suggested that the best partition scheme is16S gene/each codon position of COI gene, and selected GTR + G + I model as the best model for each partition. Phylogenetic analyses were conducted using maximum likelihood (ML) and Bayesian Inference (BI) methods, implemented in PhyML v. 3.0 (Guindon et al. 2010) and MrBayes v. 3.12 (Ronquist and Huelsenbeck 2003), respectively. For the ML tree, branch supports were drawn from 10,000 nonparametric bootstrap replicates. In BI, two runs each with four Markov chains were simultaneously run for 50 million generations with sampling every 1,000 generations. The first 25% trees were removed as the “burn-in” stage followed by calculations of Bayesian posterior probabilities and the 50% majority-rule consensus of the post burn-in trees sampled at stationarity. Finally, mean genetic distance between *Nidirana* species based on uncorrected *p*-distance model was estimated on the 16S gene using MEGA v. 6.06 (Tamura et al. 2011).

### Morphological comparisons

All ten adults (Suppl. material 1: Table S1) and one tadpole of the new species were measured. For comparisons, three adult male specimens of *N. daunchina* freshly collected from its type locality (E’mei Mountain, Sichuan Province, China) were measured (Suppl. material 1: Table S1), and measurements of *N. yaoica* were retrieved from Lyu et al. (2019a). The terminology and methods followed Fei et al. (2005), Mahony et al. (2011), and Lyu et al. (2019a). Measurements were made with a dial caliper to the nearest 0.1 mm. Twenty-four morphometric characters of adult specimens were measured:

**SVL** snout-vent length (distance from the tip of the snout to the posterior edge of the vent);

**HDL** head length (distance from the tip of the snout to the articulation of jaw);

**HDW** head width (greatest width between the left and right articulations of jaw);

**SL** snout length (distance from the tip of the snout to the anterior corner of the eye);

**ED** eye diameter (distance from the anterior corner to the posterior corner of the eye);

**IOD** interorbital distance (minimum distance between the inner edges of the upper eyelids);

**IND** internasal distance (minimum distance between the inner margins of the external nares);

**UEW** upper eyelid width (greatest width of the upper eyelid margins measured perpendicular to the anterior-posterior axis);

**TYD** maximal tympanum diameter;

**TED** tympanum-eye distance (from anterior edge of tympanum to posterior corner of the eye);

**LAL** length of lower arm and hand (distance from the elbow to the distal end of the finger IV);

**LW** lower arm width (maximum width of the lower arm);

**HND** hand length (from distal end of radioulna to tip of distal finger III);

**RAD** radioulna length (from the flexed elbow to the base of the outer palmar tubercle);

**FIL** first finger length (measured from the base of the second finger to the tip of the first finger);

**FIIL** second finger length (measured from the base of the first finger to the tip of the second);

**FIIIL** third finger length (measured from the base of the second finger to the tip of the third);

**FIVL** fourth finger length (measured from the base of the third finger to the tip of the fourth);

**HLL** hindlimb length (maximum length from the vent to the distal tip of the toe IV);

**TL** tibia length (distance from knee to tarsus);

**TW** maximal tibia width;

**THL** thigh length (distance from vent to knee);

**TFL** length of foot and tarsus (distance from the tibiotarsal articulation to the distal end of the toe IV);

**FL** foot length (distance from tarsus to the tip of the fourth toe).

The stage of the tadpole was identified following Gosner (1960). Ten morphometric characters of tadpole specimen were measured:

**TOL** total length;

**SVL** snout-vent length (distance from the tip of the snout to the posterior edge of the vent);

**BH** maximum body height;

**BW** maximum body width;

**SL** snout length (distance from the tip of the snout to the anterior corner of the eye);

**SS** snout to spiraculum (distance from spiraculum to the tip of the snout);

**IOD** interorbital distance (minimum distance between the inner edges of the upper eyelids);

**TBW** maximum width of tail base;

**TAL** tail length (distance from base of vent to the tip of tail);

**TAH** tail height (maximum height between upper and lower edges of tail).

In order to reduce the impact of allometry, the correct value from the ratio of each character to SVL was calculated and was log-transformed for subsequent morphometric analyses. Mann-Whitney *U* tests were conducted to test the significance of differences on morphometric characters between the new species, *N. daunchina*, and *N. yaoica*. The significance level was set at 0.05. Due to only the measurements SVL, HDL, HDW, SL, IND, IOD, ED, TYD, TED, HND, RAD, TL, and FL of male *N. yaoica* being available from Lyu et al. (2019a), the Mann-Whitney *U* tests were conducted based on these 13 morphometric characters for the new species and *N. yaoica*.

The new species was also compared with all other *Nidirana* species based on morphological characters. Comparative morphological data were obtained from the literature for species. *N. adenopleura* (Boulenger 1909; Chuaynkern et al. 2010; Lyu et al. 2017), *N. chapaensis* (Bourret 1937; Chuaynkern et al. 2010), *N. daunchina* (Chang and Hsü 1932; Liu 1950; Fei et al. 2009; Lyu et al. 2017), *N. hainanensis* (Fei et al. 2007; Li et al. 2019a), *N. leishanensis* (Li et al. 2019a), *N. lini* (Chou 1999; Fei et al. 2009; Lyu et al. 2017), *N. nankunensis* (Lyu et al. 2017), *N. okinavana* (Boettger 1895; Matsui 2007), *N. pleuraden* (Boulenger 1904) and *N. yaoica* (Lyu et al. 2019a).

### Bioacoustics analyses

The advertisement calls of the new *species* from Huanglian Town, Tongzi County, Guizhou Province, China were recorded from the specimen CIBTZ20190608004 in the field on 8 June 2019. The advertisement calls were recorded from the ridge of a paddy field at ambient air temperature of 20 °C and air humidity of 80 %. For comparisons, the advertisement calls of *N. daunchina* from E’mei Mountain, Sichuan Province, China were recorded from the specimen CIB2011081603 at ambient air temperature of 20 °C and air humidity of 85 % in the ridge of paddy field on 16 August 2011; the advertisement calls of *N. yaoica* were retrieved from Lyu et al. (2019a). SONY PCM-D50 digital sound recorder was used to record within 20 cm of the calling individual. The sound files in wave format were resampled at 48 kHz with sampling depth 24 bits. Calls were recorded and examined as described by Wijayathilaka and Meegaskumbura (2016). Call recordings were visualised and edited with SoundRuler 0.9.6.0 (Gridi-Papp 2003–2007) and Raven Pro 1.5 software (Cornell Laboratory of Ornithology, Ithaca, NY, USA). Ambient temperature of the type and other localities was taken by a digital hygrothermograph.

## Results

**Figure 2.**
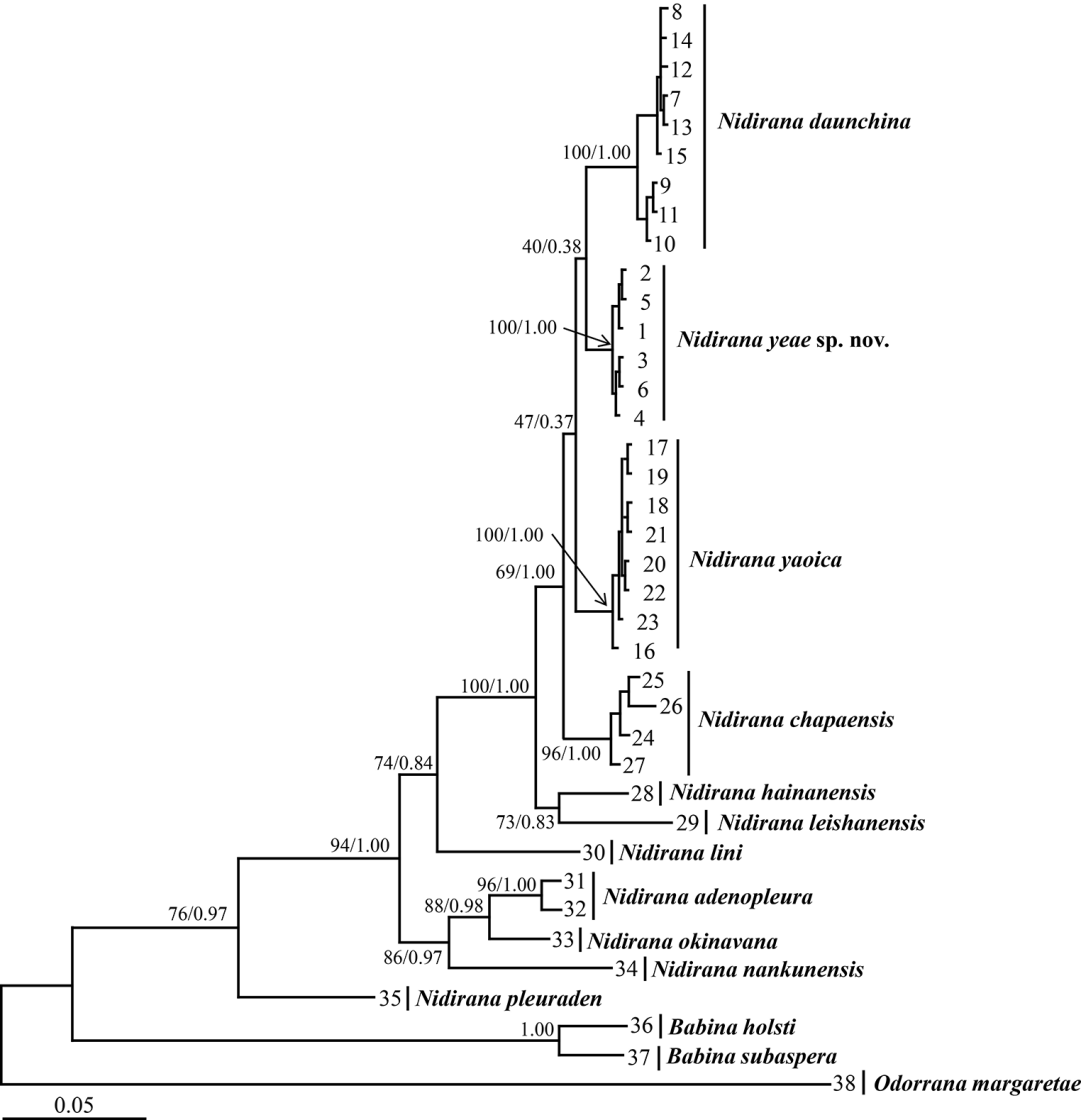
Maximum Likelihood (ML) tree based on the mitochondrial 16S and COI gene sequences. Bootstrap supports from ML analyses/Bayesian posterior probabilities from Bayesian Inference (BI) analyses are labelled beside nodes. Information of samples 1–38 in Table 1.

Aligned sequence matrix of 16S is 523 base pairs (bp) in length and 561 bp for COI. ML and BI analyses based on the 16S + COI matrix resulted in basically identical topologies (Fig. 2). All samples of the new *species* were clustered into one clade nested in the genus *Nidirana*. This new species clade was clustered into a large clade together with *N. leishanensis*, *N. hainanensis*, *N. daunchina*, *N. yaoica*, and *N. chapaensis*, with high supported value of 100 in ML and 1.00 in BI. On 16S gene, the mean genetic distance between the new species and the closely related species *N. daunchina* and *N. yaoica* were 1.2 % and 1.3 %, respectively, at the same level as the distance between *N. adenopleura* and *N. okinavana* (1.2 %; Table 2).

**Table 2.** Uncorrected *p*-distance between *Nidirana* species of the 16S rRNA gene. Mean value of genetic distance is given in the lower half of the table.

ID	Species	1	2	3	4	5	6	7	8	9	10
1	*Nidirana yeae* sp. nov.										
2	*Nidirana daunchina*	0.012									
3	*Nidirana yaoica*	0.013	0.016								
4	*Nidirana chapaensis*	0.015	0.017	0.020							
5	*Nidirana hainanensis*	0.028	0.030	0.030	0.032						
6	*Nidirana leishanensis*	0.034	0.036	0.032	0.042	0.029					
7	*Nidirana lini*	0.026	0.035	0.033	0.036	0.035	0.042				
8	*Nidirana adenopleura*	0.031	0.037	0.037	0.036	0.039	0.035	0.030			
9	*Nidirana okinavana*	0.038	0.044	0.044	0.041	0.042	0.044	0.029	0.012		
10	*Nidirana nankunensis*	0.059	0.069	0.069	0.075	0.063	0.065	0.050	0.044	0.040	
11	*Nidirana pleuraden*	0.050	0.054	0.060	0.065	0.062	0.071	0.047	0.052	0.052	0.069

The results of Mann-Whitney *U* tests indicated that in males, the new species was significantly different from *N. daunchina* and *N. yaoica* on many morphometric characters (all *p*-values < 0.05; Table 3). The new species could also be identified from its congeners based on morphological descriptions from the literature and from our examinations of newly collected material (Table 4). More detailed descriptions of results from morphological comparisons between the new species and its congeners are presented in the following sections.

**Table 3.** Morphometric comparisons between *Nidirana yeae* sp. nov., *N. daunchina* and *N. yaoica*. Units in mm. Abbreviations for the species name: NYE, *Nidirana yeae* sp. nov.; ND, *N. daunchina*; NYA, *N. yaoica*. See abbreviations for morphometric characters in Materials and methods section.

	NYE			ND		NYA		*P*-value from Mann-Whitney *U* test	
	Male (*N* = 9)		Female (*N* = 1)	Male (*N* = 3)		Male (*N* = 13)			
	Range	Mean ± SD	Value	Range	Mean ± SD	Range	Mean ± SD	NYE vs. ND	NYE vs. NYA
SVL	41.2–43.5	42.4 ± 1.8	44.7	46.1–46.3	46.2 ± 0.1	40.4–45.9	43.8 ± 1.7	0.013	0.077
HDL	14.0–15.9	15.0 ± 1.5	16.6	17.7–20.7	19.1 ± 1.5	15.7–18.6	16.9 ± 0.9	0.033	0.021
HDW	14.4–15.5	15.0 ± 0.8	15.1	16.2–17.5	16.8 ± 0.6	15.0–17.2	16.0 ± 0.6	0.309	0.025
SL	6.5–7.0	6.8 ± 0.5	7.1	7.0–7.3	7.2 ± 0.2	6.2–8.7	7.2 ± 0.7	0.116	0.92
IND	5.2–5.6	5.4 ± 0.3	5.6	5.5–6.6	6. 0 ± 0.5	5.4–6.6	5.9 ± 0.3	0.926	0.102
IOD	4.2–4.7	4.5 ± 0.4	4	4.2–4.8	4.4 ± 0.3	3.5–5.1	4.3 ± 0.5	0.644	0.92
ED	3.9–4.6	4.2 ± 0.6	5.1	5.4–6.2	5.8 ± 0.4	4.6–5.4	5.1 ± 0.2	0.033	0.015
UEW	2.8–3.4	3.1 ± 0.5	2.4	3.1–3.3	3.2 ± 0.1	/	/	0.309	/
TYD	3.6–4.2	3.9 ± 0.4	3.7	4.0–4.8	4.4 ± 0.4	3.2–4.5	3.9 ± 0.4	0.926	0.526
TED	1.2–2.0	1.5 ± 0.3	1.6	0.8–1.2	1.0 ± 0.2	1.0–1.6	1.2 ± 0.2	0.013	0.018
LAL	16.9–18.2	17.5 ± 1.0	19.1	19.8–21.1	20.4 ± 0.7	/	/	0.079	/
LW	3.6–3.9	3.8 ± 0.2	3.9	3.6–4.6	4.1 ± 0.5	/	/	0.782	/
HND	10.1–11.9	11.0 ± 0.5	11	11.4–12.1	11.8 ± 0.4	10.2–12.8	11.1 ± 0.9	0.782	0.367
RAD	7.7–9.6	8.6 ± 0.7	9	9.7–9.9	9.8 ± 0.1	7.8–9.4	8.5 ± 0.4	0.166	0.018
FIL	5.0–6.0	5.5 ± 0.4	6	5.7–6.6	6.1 ± 0.4	/	/	0.926	/
FIIL	3.3–4.6	4.1 ± 0.4	4.4	4.3–4.9	4.7 ± 0.3	/	/	0.309	/
FIIIL	5.8–7.6	6.8 ± 0.5	7.1	6.2–7.5	6.9 ± 0.6	/	/	0.405	/
FIVL	4.5–5.0	4.7 ± 0.2	5	4.5–5.3	5.0 ± 0.4	/	/	0.926	/
HLL	62.7–67.4	65.0 ± 3.7	62.4	73.6–75.1	74.3 ± 0.7	/	/	0.033	/
THL	19.6–21.4	20.6 ± 1.5	21.7	20.6–23.0	21.9 ± 1.2	/	/	0.782	/
TL	20.9–22.1	21.5 ± 1.0	22.6	23.8–24.3	24.0 ± 0.3	21.6–25.6	23.1 ± 1.0	0.405	0.018
TW	6.4–6.9	6.6 ± 0.4	6	5.9–7.0	6.6 ± 0.6	/	/	0.033	/
TFL	28.7–30.9	29.8 ± 1.9	33.2	23.9–24.9	24.2 ± 0.6	/	/	0.013	/
FL	21.3–22.7	21.9 ± 1.1	23.3	34.3–36.7	35.4 ± 1.2	31.1–35.7	31.4 ± 9.0	0.013	0.001

**Table 4.** Diagnostic characters separating *Nidirana yeae* sp. nov. from its congeners.

Species	SVL of male (mm)	SVL of female (mm)	Fingers tips	Lateroventral groove on fingers	Relative finger length	Toe tips	Lateroventral groove on toes	Tibiotarsal articulation reaching level when leg stretched forward	Subgular vocal sacs	Nuptial pad	Tadpole labial tooth row formula	Calling	References
*Nidirana yeae* sp. nov.	41.2–43.5	44.7	dilated	absent	II < IV < I < III	dilated	present	eye	present	one on first finger	1:1+1/1+1:2	2–6 notes	This study
*N. adenopleura*	43.1–57.6	47.6–60.7	dilated	present or absent	II *<* I *<*IV *<* III	dilated	present	snout tip or between eye and snout	present	one on first finger	1:1+1/1+1:2 or 1:0 +0/1+1:1	2–4 notes	Pope (1931); Chuaynkern et al. (2010); Lyu et al. (2017)
*N. chapaensis*	35.5–42.5	41.0–51.8	dilated	present or absent	II *<* I = IV *<* III	dilated	present	nostril	present	two on first finger	1:1+2/1+1:2	3 notes	Chuaynkern et al. (2010)
*N. daunchina*	40.6–51.0	44.0–53.0	dilated	present	II < IV < I < III	dilated	present	nostril	present	one on first finger	1:1+1/1+1:2 or 1:1+1/2+2:1	2–5 notes containing a specific first note	This study; Liu (1950); Fei et al. (2009); Lyu et al. (2017)
*N. hainanensis*	32.8–33.5	/	dilated	present	II *<*I *<* IV *<* III	dilated	present	nostril	present	absent	/	2–4 fast-repeated double notes	Fei et al. (2009); Lyu et al. (2017); Li et al. (2019a)
*N. leishanensis*	49.5–56.4	43.7–55.3	dilated	present	II < IV < I < III	dilated	present	between eye and snout	present	two on first two finger	1:1+2/1+1: 2	1 note	Li et al. (2019a)
*N. lini*	44.1–63.1	57.7–68.6	dilated	present or absent	II *<* I *<* IV *<* III	dilated	present	beyond snout	present	one on first finger	1:1+1/1+1:2	5–7 notes	Chou (1999); Fei et al. (2009); Lyu et al. (2017)
*N. nankunensis*	33.3–37.1	37.8–39.5	dilated	present or absent	II *<* I *<* IV *<* III	dilated	present	nostril	present	one on first finger	1:1+1/1+1:2	13–15 fast-repeated notes	Lyu et al. (2017)
*N. okinavana*	35.5–42.8	44.6–48.8	dilated	present or absent	II *<* I *<* IV *<* III	dilated	present	between eye and nostril	absent	poorly one on first finger	1:1+1/1+1:2	17–25 fast-repeated notes	Matsui and Utsunomiya (1983); Chuaynkern et al. (2010)
*N. pleuraden*	45.4–58.7	45–62.5	not dilated	absent	II *<* I *<* IV *<* III	not dilated	absent	between eye and snout	present	one on first finger	1:1+1/1+1:2 or 1:1+1/2+2:1	4–7 notes	Lyu et al. (2017)
*N. yaoica*	40.4–45.9	/	dilated	present	II *<* I *<* IV *<* III	dilated	present	nostril	present	one on first finger	/	1–3 fast-repeated notes	Lyu et al. (2019a)

**Figure 3.**
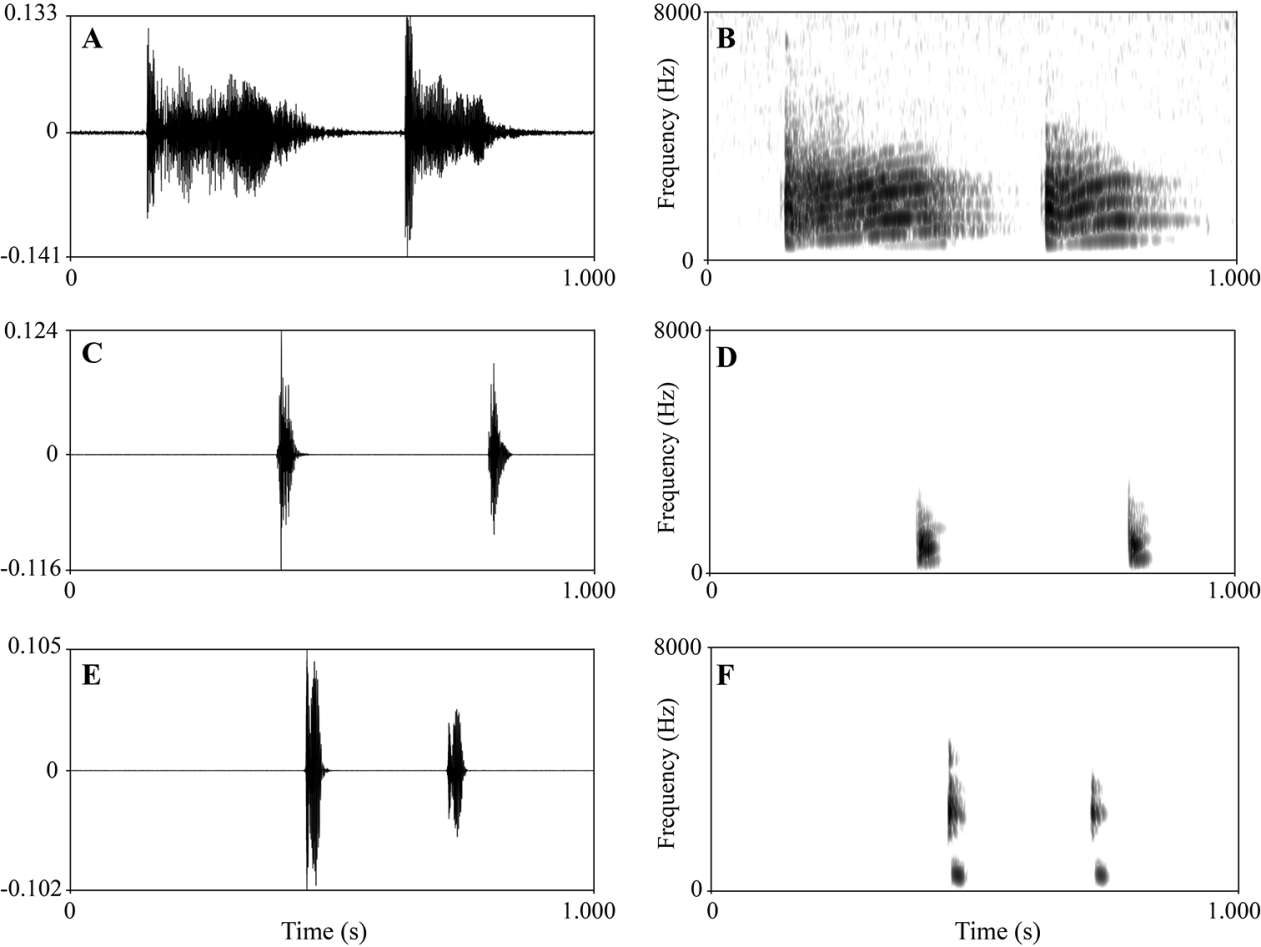
Advertisement calls of *Nidirana yeae* sp. nov. (holotype CIBTZ20190608004), *N. daunchina* (specimen CIB2011081603) and *N. yaoica* (specimen SYS a007009). **A** Waveform showing two-note call of *Nidirana yeae* sp. nov **B** sonogram showing two-note call of *Nidirana yeae* sp. nov **C** waveform showing two-note call of *N. daunchina***D** sonogram showing two-note call of *N. daunchina***E** waveform showing two-note call of *N. yaoica***F** sonogram showing two-note call of *N. yaoica*.

There were many differences in sonograms and waveforms of calls between the new species, *N. daunchina*, and *N. yaoica* (Fig. 3; Table 5). Firstly, in the call duration and the note duration, the two-note call and three-note call of the new species were longer than those of both *N. daunchina* and *N. yaoica*. Secondly, the note interval of two-note call and three-note call of the new species was shorter than those of *N. daunchina* and *N. yaoica*. Thirdly, the dominant frequency of call in the new species was higher than *N. yaoica*.

**Table 5.** Comparisons of characteristics of advertisement calls of *Nidirana yeae* sp. nov., *N. daunchina*, and *N. yaoica*. Units in milliseconds (ms).

*Nidirana yeae* sp. nov.				*N. daunchina*		*N. yaoica*		
Two-note call (N = 5)	Three-note call (*N* = 2)	Four-note call (*N* = 3)	Six-note call (*N* = 1)	Two-note call (*N* = 5)	Three-note call (*N* = 2)	One-note call (*N* = 25)	Two-note call (*N* = 59)	Three-note call (*N* = 3)
728–825, 755.4 ± 45.2	988–1135, 1061.5 ± 103.9	1400–1563, 1459.3 ± 90.0	2082	453–462, 457.7 ± 4.5	768–826, 792.0 ± 21.1	37–51, 43.3 ± 2.7	307–454, 355.9 ± 31.1	565–678, 628.0 ± 57.6
1^st^ 342–418, 362.0 ± 31.8	1^st^ 308–443, 375.5 ± 95.4	1^st^ 314–403, 364.0 ± 45.5	1^st^ 440	1^st^ 45–65, 56.0 ± 10.1	1^st^ 43–55, 52.0 ± 5.1	1^st^ 37–51, 43.3 ± 2.7	1^st^ 36–51, 43.5 ± 2.8	1^st^ 42–54, 46.7 ± 6.4
2^nd^ 212–225, 218.6 ± 6.1	2^nd^ 169–220, 194.5 ± 36.1	2^nd^ 203–218, 212.0 ± 79.4	2^nd^ 240	2^nd^ 47–53, 49.3 ± 3.2	2^nd^ 49–60, 55.0 ±4.7		2^nd^ 30–49, 39.6 ± 3.3	2^nd^ 37–40, 38.7 ± 1.5
	3^rd^ 135–205, 170.0 ± 49.5	3^rd^ 166–180, 170.6 ± 8.1	3^rd^ 194		3^rd^ 38–58, 45.0 ± 7.9			3^rd^ 35–52, 42.3 ± 8.7
		145–172, 157.0 ± 13.7	4^th^ 175					
			5^th^ 160					
			6^th^ 166					
151–197, 170.0 ± 19.1	1^st^ 120–194, 157.0 ± 52.3	1^st^ 175–218, 194.6 ± 21.7	1^st^ 132	347–359, 352.0 ± 6.2	1^st^ 320–355, 337.0 ± 15.2	/	215–372, 272.8 ± 31.7	1^st^ 212–250, 234.0 ± 19.7
	2^nd^ 147–178, 162.5 ± 21.9	2^nd^ 155–185, 174.0 ± 16.5	2^nd^ 132		2^nd^ 298–310, 303.0 ± 4.9			2^nd^ 222–302, 266.3 ± 40.7
		3^rd^ 138–228, 190.7 ± 46.9	3^rd^ 135					
			4^th^ 126					
			5^th^ 132					
4200–5040, 4776.0 ± 332.9	4620–5040, 4830.0 ± 296.9	4680–5160, 4880.0 ± 249.7	5280	3629–4240, 3938.0 ± 305.6	3875–4832, 4586.4 ± 402.0	516.8	516.8	516.8
1^st^ 4200–4800, 4440.0 ± 226.2	1^st^ 4320–4440, 4380.0 ± 84.8	1^st^ 4680–5160, 4880.0 ± 249.7	1^st^ 4560	1^st^ 3629–4240, 3899.3 ± 311.5	1^st^ 2624–4448, 3894.4 ± 774.7	516.8	1^st^ 516.8 (98.3%) or 2584 (1.7%)	1^st^ 516.8
2^nd^ 4200–5040, 4776.0 ± 332.9	2^nd^ 4620–5040, 4830 ± 297	2^nd^ 4080–4680, 4400.0 ± 301.9	2^nd^ 5280	2^nd^ 2151–3945, 3187.6 ± 929.0	2^nd^ 3875–4832, 4586.4 ± 402.0			2^nd^ 516.8
	3^rd^ 3840–4560, 4200.0 ± 509.1	3^rd^ 4080–4680, 4440.0 ± 317.5	3^rd^ 4800		3^rd^ 1478–3200, 2241.2 ± 662.8		2^nd^ 516.8	3^rd^ 516.8
		4^th^ 4320–4680, 4466.6 ± 189.0	4^th^ 4560					
			5^th^ 3800					
			6^th^ 4080					

Based on the molecular, morphological, and bioacoustics differences, the specimens from Tongzi County, Guizhou Province, China, represent a new species which is described as *Nidirana yeae* sp. nov.

### Taxonomic account

#### Nidirana yeae sp. nov.

Taxon classificationAnimaliaAnuraRanidae

42301A4E-5191-51B7-B15C-9556E9149DBC

http://zoobank.org/B4E44DFA-4720-4B28-8CF1-DEFC6096D2EF

**Figure 4.**
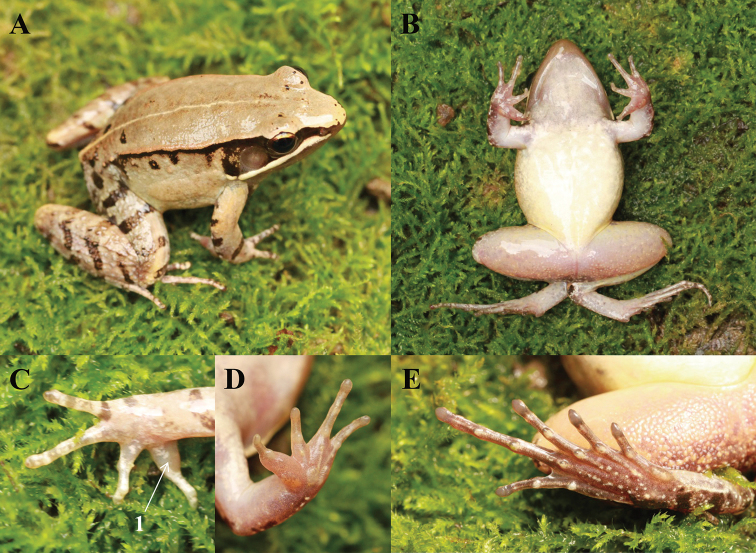
Photos of the holotype CIBTZ20190608004 of *Nidirana yeae* sp. nov. in life. **A** Dorsal view **B** ventral view **C** dorsal view of hand **D** ventral view of hand **E** ventral view of foot. Key: 1 indicates nuptial pad on the inner side of finger I.

**Figure 5.**
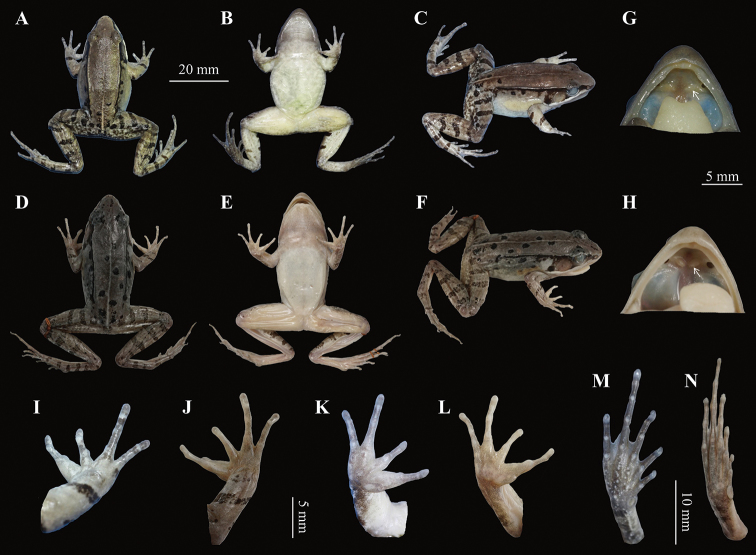
The holotype specimen CIBTZ20190608004 of *Nidirana yeae* sp. nov. and topotype specimen CIB2011081603 of *N. daunchina*. **A–C** Dorsal view, ventral view and dorsolateral view of CIBTZ20190608004 **D–F** dorsal view, ventral view and dorsolateral view of CIB2011081603 **G, H** oral cavity of CIBTZ20190608004 and CIB2011081603 (arrow point to vomerine ridge) **I, J** dorsal view of hand of CIBTZ20190608004 and CIB2011081603 **K, L** ventral view of hand of CIBTZ20190608004 and CIB2011081603 **M, N** ventral view of foot of CIBTZ20190608004 and CIB2011081603.

**Figure 6.**
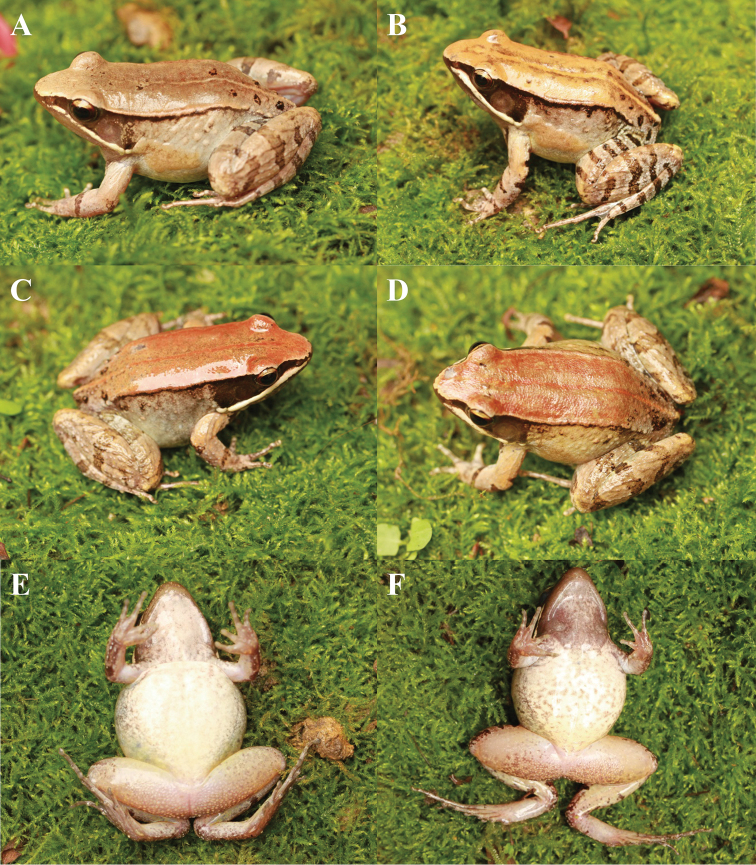
Colour variation in *Nidirana yeae* sp. nov. **A** Dorsal view of male specimen CIBTZ20190608003 **B** dorsal view of male specimen CIBTZ20190608016 **C** dorsal view of female specimen CIBTZ20190608005 **D** dorsal view of male specimen CIBTZ20190608006 **E** ventral view of male specimen CIBTZ20190608006 **F** ventral view of female specimen CIBTZ20190608005.

Figures 4 , 5A–C , 6 ; Table 1 , Suppl. material 1: Table S1

##### Material examined.

***Holotype.*** CIBTZ20190608004 (Figs 4, 5), adult male, collected by Shi-Ze Li on 6 June 2019 in Huanglian Town (28.44317N, 107.02003E; ca. 1170 m a.s.l.), Tongzi County, Guizhou Province, China.

***Paratypes.*** A total of nine specimens (eight adult males and one adult female) collected by Shi-Ze Li from Huanglian Town in Tongzi County, Guizhou Province, China. Two male specimens: CIBTZ20160714016 and CIBTZ20160714017 collected on 14 July 2016; one female specimen: CIBTZ20190608005 and six male specimens: CIBTZ20190608001, CIBTZ20190608003, CIBTZ20190608006, CIBTZ20190608010, CIBTZ20190608011, CIBTZ20190608013, CIBTZ20190608016 and CIBTZ20190608017 collected on 8 June 2019.

##### Other material examined.

One tadpole (CIBTZ20190608019) collected by Jing Liu on 8 June 2019.

##### Diagnosis.

*Nidirana yeae* sp. nov. is assigned to the genus *Nidirana* based on molecular data and the following combination of characters: absence of thumb-like structure on finger I; disks of digits dilated, rounded; dorsolateral folds distinct; the presence of large suprabrachial gland in male.

*Nidirana yeae* sp. nov. could be distinguished from its congeners by a combination of the following characters: (1) body of medium size (SVL 41.2–43.5 mm in males and 44.7 mm in female); (2) lateroventral groove only present on toes; (3) relative finger lengths: II < IV < I < III; (4) three metatarsal tubercles on palm; (5) heels overlapping when hindlimbs flexed at right angles to axis of body; (6) tibiotarsal articulation reaching the level of eye when leg stretched forward; (7) a pair of subgular internal vocal sacs at corners of throat in male; (8) nuptial pad present on the inner side of base of fingers I in male in breeding season; (9) tadpole labial tooth row formula with 1:1+1/1+1:2; (10) in male, the advertisement call containing two kinds of note and the call containing 2–6 repeated regular notes.

##### Description of holotype.

Body size medium, SVL 40.2 mm; head slightly wider than long (HDW/HDL = 1.03), flat above; snout rounded in dorsal and lateral views, slightly projecting beyond lower jaw; a maxillary gland in posterior corner of mouth from snout to tympanum, behind the gland a shoulder gland present; supratympanic fold absent; interorbital space narrower than internarial distance (IND/IOD = 1.38); eye large and convex, ED 0.76 times of SL; tympanum distinct, large and rounded, 0.76 times of ED, and close to eye; vomerine ridge present, but the outline of vomerine ridges are not sharp and almost connected to the internal nostril; tongue deeply notched posteriorly; paired subgular inner vocal sacs at corners of throat.

Forelimbs moderately robust (LW/SVL = 0.08); lower arm and hand less than a half of body length (LAL/SVL = 0.42); relative finger lengths: II < IV < I < III; tip of fingers weakly dilated, forming elongated and pointed disks; lateroventral grooves on the disks of finger absent; fingers free of webbing, with lateral fringes on fingers III and IV; subarticular tubercles prominent and rounded; week supernumerary tubercles below the base of fingers III and IV; palmar tubercles three, elliptic, distinct.

Hindlimbs relatively robust, tibia 47% of SVL; tibia longer than thigh (TL/THL = 1.04); heels overlapping when hindlimbs held at right angles to axis of body; tibiotarsal articulation reaching the level of mid-eye when hindlimb is stretched forward; toes long and thin, relative toe lengths: I < II < V < III < IV; tip of toes dilated, forming significantly elongated disks; distinct lateroventral grooves on toes; webbing weak, webbing formula:

$I2-2\mathrm{II}1\frac{2}{3}-3\frac{1}{2}\mathrm{III}2\frac{1}{2}-3\frac{2}{3}\mathrm{IV}3\frac{2}{3}-2V$;

toes with lateral fringes; subarticular tubercles rounded, prominent; inner metatarsal tubercle elliptic, twice as long as its width; outer metatarsal tubercle indistinct, small and rounded.

Dorsal skin of head and anterior part of body smooth, posterior part and flanks with several tubercles, some tubercles with black spot; a large suprabrachial gland behind base of forelimb; dorsolateral fold extending from posterior margin of upper eyelid to above groin; several granules on the dorsal surfaces of thigh, tibia, and tarsus; ventral surface of head, body, and limbs smooth, several flattened tubercles densely arranged on the rear of thigh and around vent.

##### Colouration of holotype in life.

In life, dorsal surface and suprabrachial gland pale brown; ﬂank relatively smooth with dense tubercles on region nearly the dorsolateral fold; several black spots on flank, dorsum, and head; a discontinuous light yellow streak from posterior head to cloacae; dorsal forelimbs light brown and one brown stripe in front of the base of forelimb; dorsal hindlimb grey-brown with dense tubercles, three brown bands on the thigh, four on the tibia and the tarsus; tympanum and temporal region black; maxillary gland white; ventral surface smooth, throat and ventral of thigh and forelimbs incarnadine, belly and chest light yellow (Fig. 4).

##### Preserved holotype colouration.

Dorsal surface faded to brown; black spots on dorsum and flank more distinct; limbs faded light brown and the crossbars becoming clearer; ventral surface faded to pale cream and throat fade to brownness (Fig. 5).

##### Variations.

All adult specimens were similar in morphology but some individuals differed from the holotype in colour pattern. In some adult males, the colour of tympanum and temporal region pinkish red (Fig. 6A); in some adult males, the colour of dorsum is reddish brown (Fig. 6B); in the adult female, the colour of dorsum was brownish red and the flank was brownish under the dorsolateral fold (Fig. 6C); in some adult males, the colour of dorsum brick-red and the tubercles on flank were obvious (Fig. 6D); in some adult males, the throat was creamy and ventral surface of body was white with brown patches (Fig. 6E); in the adult female, the throat was brown and there were some patchiness on the ventral surface of the body and thigh (Fig. 6F).

##### Tadpole description.

**Figure 7.**
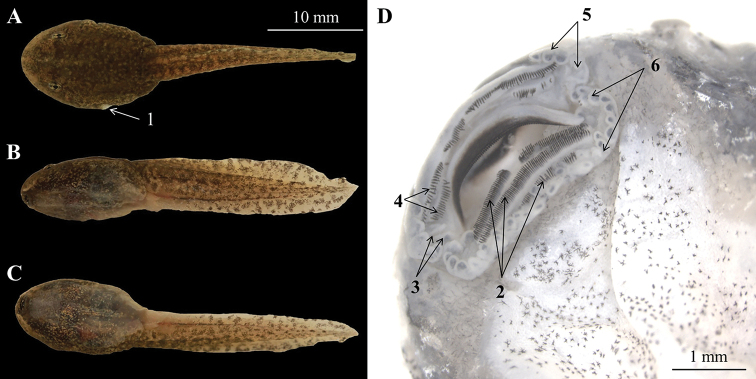
The tadpole CIBTZ20190608019 of *Nidirana yeae* sp. nov. in life. **A** Dorsal view **B** lateral view **C** ventral view **D** mouth structure. Key: **1**, spiracle; **2**, lower keratodonts; **3**, additional tubercles at the angles of mouth; **4**, upper keratodonts; **5**, labial papillae on upper lips; **6**, labial papillae on lower lips.

Measurements of specimen CIBTZ20190608019 (in mm): TOL 35.2, SVL 14.0, BW 6.1, BH 5.1, SL 3.1, SS 8.1, IOD 3.3, TAL 20.7, TAH 4.0, TBW 3.0. Body oval, body and tail yellowish brown, flattened above; several brown spots on dorsum and tail; maximum depth near posterior part of tail and more than body depth; body width longer than body height (BW/ BH = 1.53); eyes lateral, nostril near snout; spiracle on left side of body, directed dorsoposteriorly; keratodont formula: 1:1+1/1+1:2; ventral of body oval, creamy white with dense brown spots on flank of body; both upper and lower lips with labial papillae; some additional tubercles at the angles of the mouth, usually with small keratodonts; tail fusiform, approximately 1.5 times as long as snout-vent length, tail height 19.3 % of tail length; dorsal fin arising behind the origin of the tail (Fig. 7).

##### Advertisement call.

Eleven advertisement calls of *Nidirana yeae* sp. nov. were recorded from the holotype CIBTZ20190608004 on the ridge of a paddy field in Huanglian Town, Tongzi County, Guizhou Province, China on 8 June 2019 between 21:00–22:00. The call has two kinds of notes (Fig. 3; Table 5). Call duration was 728–2082 ms (mean 1199 ± 174 ms, *N* = 11). Call interval was 2000–9435 ms (mean 4586 ± 2659 ms, *N* = 10). The first type of note is the start note in each call and the other notes in each call are termed the second type. Amplitude modulation within strophe is apparent, beginning with moderate energy pulses, decreasing slightly to a minimum then increasing approximately to the midnote, subsequently increasing to a peak then decreasing rapidly towards the end of each note in the first type; in the second type amplitude beginning with highest pulses and decreasing towards approximately the midnote then increasing slightly then decreasing towards the end of each note. The first type of note has a longer duration than the second type (308–440 ms, *N* = 10 vs.135–240 ms, *N* = 23).The two-note call (*N* = 5) has a duration of 728–825 ms, and dominant frequency is 4200–5040 Hz, three-note call (*N* = 2) has a duration of 988–1135 ms and dominant frequency is 4620–5040 Hz, four-note call (*N* = 3) has a duration of 1400–1563 ms and dominant frequency is 4680–5160 Hz, six-note call (*N* = 1) has a duration of 2082 ms and dominant frequency is 5280 Hz (Table 5).

##### Secondary sexual characteristics.

A pair of subgular inner vocal sacs, a pair of slit-like openings at posterior of jaw; a single light brown nuptial pad on the inner side of dorsal surface of finger I (Fig. 4C); nuptial spicules invisible; suprabrachial gland present.

##### Morphological comparisons.

*Nidirana yeae* sp. nov. differs from *N. leishanensis* and *N. lini* by having smaller body size (SVL < 45 mm in the new species vs. SVL > 49 mm in males of *N. leishanensis* and SVL > 57 mm in females of *N. lini*).

*Nidirana yeae* sp. nov. differs from *N. daunchina*, *N. hainanensis* and *N. leishanens* by the presence of lateroventral groove only on toes (vs. both fingers and toes present in the latter).

*Nidirana yeae* sp. nov. differs from *N. pleuraden* by the presence of lateroventral groove only on toes (vs. both fingers and toes absent in the latter).

*Nidirana yeae* sp. nov. differs from *N. adenopleura*, *N. hainanensis*, *N. lini*, *N. nankunensis*, *N. okinavana*, and *N. pleuraden* by the relative finger lengths II < IV < I < III (vs. II < I < IV < III or II < I = IV < III in the latter).

*Nidirana yeae* sp. nov. differs from *N. hainanensis*, *N. lini*, and *N. nankunensis* by tibiotarsal articulation reaching the level of eye when leg stretched forward (vs. reaching nostril or beyond snout in the latter).

*Nidirana yeae* sp. nov. differs from *N. okinavana* by having subgular internal vocal sacs (vs. gular vocal sacs absent in the latter).

*Nidirana yeae* sp. nov. differs from *N. hainanensis* and *N. leishanensi* by having nuptial pad on the inner side of base of fingers I in males in breeding season (vs. nuptial pad absent in *N. hainanensis* and nuptial pads on both fingers I and II in *N. leishanensis*).

*Nidirana yeae* sp. nov. differs from *N. nankunensis* and *N. okinavana* by the call containing 2–6 notes (vs. 13–15 notes in *N. nankunensis* and 17–25 notes in *N. okinavana*).

*Nidirana yeae* sp. nov. is genetically closer to *N. chapaensis*, *N. daunchina*, and *N. yaoica*. It differs from *N. chapaensis* by the following characters: the relative finger lengths II < IV < I < III (vs. II < I = IV < III), tibiotarsal articulation reaching the level of eye when leg stretched forward (vs. reaching nostril), having nuptial pad on the inner side base of finger I in males in breeding season (vs. having two nuptial pads on finger I), tadpole labial tooth row formula of 1:1+1/1+1:2 (vs. 1:1+2/1+1:2); differs from *N. daunchina* by the presence of lateroventral groove only on toes (vs. both fingers and toes present), heels overlapping when hindlimbs flexed at right angles to axis of body (vs. heels meeting), tibiotarsal articulation reaching the level of eye when leg stretched forward (vs. reaching nostril), having significantly lower value of SVL in males and having significantly lower ratios of HDL, ED, TED, HLL, TW, TFL, and FL to SVL in males, the outline of vomerine ridges not sharp and almost connected to the internal nostril (vs. outline of vomerine ridges sharp and distinctly separated from the internal nostril; Fig. 5G, H), having longer call duration in two-note call and three-note call, having shorter note interval in the two-note call and three-note call (Table 4); differs from *N. yaoica* by the presence of lateroventral groove only on toes (vs. both fingers and toes present), relative finger lengths II < IV < I < III (vs. II < I < IV < III), tibiotarsal articulation reaching the level of eye when leg stretched forward (vs. reaching nostril), having significantly lower ratios of HDL, HDW, ED, TED, RAD, TL, and FL of SVL in males, having longer call duration and longer note duration in two-note call and three-note call, having shorter note interval in two-note call and three-note call, and having higher dominant frequency in call (Table 5).

##### Remarks.

Wu et al. (1986) reported that the populations from Kuankuoshui Nature Reserve of Suiyang County, Fanjiang Mountain of Jiangkou County and Leigong Mountain of Leishan County, Guizhou Province, China belonged to *N. adenopleura*. Fei et al. (1990, 2009) suggesting that populations from Kuankuoshui Nature Reserve of Suiyang County together with the populations from north-eastern part of Guizhou Province, China should be *N. daunchina*, and the populations from Fanjiang Mountain of Jiangkou County and Leigong Mountain of Leishan County, Guizhou Province, China should be *N. adenopleura*. Li et al. (2019a) proved that the population from Leigong Mountain of Leishan County should be a new species, which they named *N. leishanensis*. From the morphological description and morphometric data of the population from Kuankuoshui Nature Reserve of Suiyang County, some characters is very similar to *Nidirana yeae* sp. nov.: body of medium size (SVL 39.0–46 mm in males and 44–48 mm in females); lateroventral groove on toes present; relative finger lengths II < IV < I < III; three metatarsal tubercles on palm; a pair of subgular internal vocal sacs at corners of throat in males; nuptial pad present on the inner side of base of fingers I in males in breading season; tadpole labial tooth row formula with 1:1+1/1+1:2. This population was probably *Nidirana yeae* sp. nov., and detailed comparisons especially with molecular data should be conducted to establish its identity. The phylogenetic trees in our work and Lyu et al. (2019a) all supported that the population from Hejiang County, Sichuan Province, China was the closest to topotypes of *N. daunchina* but separated from *Nidirana*yeae sp. nov. and other relatives, and so this population in the south-eastern part of Sichuan Province should be *N. daunchina* and not *Nidirana yeae* sp. nov. Although the morphometric data (large body size) of the population from Fanjiang Mountain of Jiangkou County in Wu et al. (1986) indicated that it was similar to *N. leishanensis*, we still need detailed comparisons and molecular data to clarify its taxonomic status.

##### Ecology.

**Figure 8.**
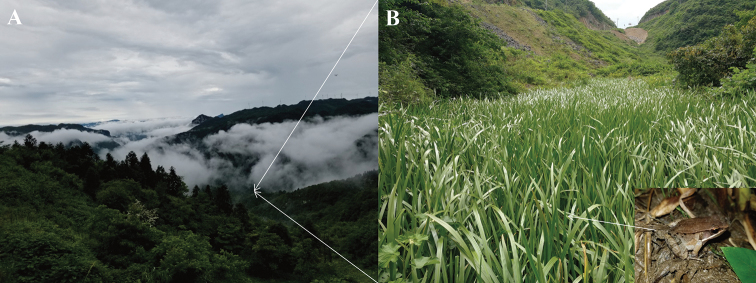
Habitats of *Nidirana yeae* sp. nov. in the type locality, Huanglian Town, Tongzi County, Guizhou Province, China. **A** Landscape of montane forests in the type locality **B** a paddy field occupied by the species in its type locality. *insert*: a male of *Nidirana yeae* sp. nov. in the paddy field.

*Nidirana yeae* sp. nov. is currently found from the paddy field (28.44317N, 107.02003E; ca. 1170 m a. s. l.) in Huanglian Town, Tongzi County, Guizhou Province, China. The individuals were found on the paddy field near an evergreen broad-leaved forest (Fig. 8). Tadpoles of the species could be found in the water. Two sympatric amphibians, *Zhangixalus omeimontis* (Stejneger, 1924) and *Polypedates braueri* (Vogt, 1911) were also found in the type locality.

##### Etymology.

The specific name *yeae* is in homage to the famous taxonomist Ye Chang-Yuan for her great contributions to Chinese amphibian research. For the common name, we suggest Ye’s Music Frog (English) and Ye Shi Qin Wa (Chinese).

## Discussion

Before this work, the taxonomic status for the populations of *Nidirana* in Tongzi County in the north part of Guizhou Province had not been reported, but populations in Suiyang County adjacent to Tongzi County were identified as *N. daunchina* (Fei et al. 1990, 2009, 2012; Fei and Ye 2005). Now, the Tongzi population is revealed as a new species, *Nidirana yeae* sp. nov. based on integrative taxonomy using morphological comparisons, molecular phylogenetic analyses, and bioacoustics. *Nidirana* populations in Suiyang County, to the east of Tongzi County, are probably the new species also. Moreover, Lyu et al. (2017) recognised three specimens (included in our phylogenetic analyses) from Hejiang County, Sichuan Province as *N. daunchina*; of note, the straight-line geographical distance between Hejiang County and Tongzi County is ca. 110 km, much shorter than that (ca. 280 km) between Hejiang County and the type locality of *N. daunchina* (E’mei Mountain, Sichuan Province, China). Therefore, it could be speculated that the two closely related species, *Nidirana yeae* sp. nov. and *N. daunchina*, were probably parapatric in the region between Hejiang and Tongzi counties. Many more surveys of the surrounding areas are needed to clarify the populations of “*N. daunchina*” and the accurate distribution of the two species.

South-western China has long been proposed as biodiversity hotspot (Myers et al. 2000). However, Guizhou Province is an important part of south-western China, especially with the particular environments of karst rocky desertification, and knowledge of biodiversity levels and/or patterns are still seriously lacking. Recently, a series of new amphibian species were described from this province (Zhang et al. 2017; Li et al. 2018a, b, 2019a, b; Lyu et al. 2019b; Wang et al. 2019), indicating that species diversity of amphibians in this region is highly underestimated. It is urgent for herpetologists to conduct comprehensive and in-depth surveys to discover the level of amphibian species diversity in this region under accelerating global changes.

## Supplementary Material

XML Treatment for Nidirana yeae

## Supplementary materials

Supplementary material 1 Table S1. Measurements of *Nidirana yeae* sp. nov. and *N. daunchina* Data type: species data Explanation note: Units in mm. See abbreviations for characters in the Materials and methods section. https://binary.pensoft.net/file/372401This dataset is made available under the Open Database License (http://opendatacommons.org/licenses/odbl/1.0/). The Open Database License (ODbL) is a license agreement intended to allow users to freely share, modify, and use this Dataset while maintaining this same freedom for others, provided that the original source and author(s) are credited.Gang Wei, Shi-Ze Li, Jing Liu, Yan-Lin Cheng, Ning Xu, Bin Wang
